# LncRNA TUG1 Regulates Proliferation of Cardiac Fibroblast *via* the miR-29b-3p/TGF-β1 Axis

**DOI:** 10.3389/fcvm.2021.646806

**Published:** 2021-09-03

**Authors:** Yini Guo, Zongli Sun, Minghe Chen, Junjie Lun

**Affiliations:** ^1^First Department of Cardiology, Changle People's Hospital, Weifang, China; ^2^Second Department of Cardiology, Changle People's Hospital, Weifang, China; ^3^Department of Oncology, Changle People's Hospital, Weifang, China

**Keywords:** atrial fibrillation, cardiac fibroblasts, proliferation, TUG1, miR-29b-3p/TGF-β1

## Abstract

**Background:** Atrial fibrillation (AF) is a very common clinical arrhythmia, accompanied by the overproliferation of cardiac fibroblasts (CFs). This study aimed to investigate the role of the long non-coding RNA(lncRNA) taurine upregulated gene 1 (TUG1) in the proliferation of CFs and further investigated its underlying mechanism.

**Methods:** One hundred four paroxysmal AF patients and 94 healthy controls were recruited. Human cardiac fibroblasts (HCFs) were applied to establish an AF cell model through treatment with angiotensin II (AngII). qRT-PCR was used for the measurement of gene levels. The cell proliferation was detected by cell counting kit-8 (CCK-8). Luciferase reporter assay was performed for target gene analysis.

**Results:** Elevated levels of TUG1 and low expression of miR-29b-3p were detected in the serum of AF patients compared with the healthy controls. Pearson's correlation analysis exhibited an inverse relationship between TUG1 and miR-29b-3p expression in AF patients (*r* = −7.106, *p* < 0.001). Knockdown of TUG1 inhibited AngII-induced CF proliferation. Taurine upregulated gene 1 (TUG1) functions as a competing endogenous RNA (ceRNA) for miR-29b-3p, and downregulation of miR-29b-3p reversed the role of TUG1 in CF proliferation. TGF-β1 is a direct target gene of miR-29b-3p.

**Conclusions:** Long non-coding RNA taurine upregulated gene 1 is a key regulator in the occurrence of AF. Slicing TUG1 inhibits CF proliferation by regulating the miR-29b-3p/TGF-β1 axis.

## Introduction

Atrial fibrillation (AF) is a very common clinical arrhythmia and increases with the growth of age. The increase in the prevalence of AF is closely related to the high incidence of hypertension, coronary heart disease, and other heart diseases (1). The implications caused by AF, such as cardiac output, thromboembolism, stroke, and heart failure, pose a serious threat to human health (2). Myocardial fibrosis is the pathophysiological basis of many cardiovascular diseases including AF and is an important feature of myocardial remodeling (3). Atrial fibrosis is the most prominent manifestation of structural remodeling in the pathology of AF (4). During the development of myocardial fibrosis, the proliferation of cardiac fibroblasts (CFs) is particularly prominent (5). Therefore, inhibiting the proliferation of CFs becomes the key point of inhibiting myocardial fibrosis.

Long non-coding RNAs (lncRNAs) are non-coding RNAs with a length of more than 200 nucleotides. Long non-coding RNAs can regulate the expression of genes at the transcriptional and post-transcriptional levels and play an important role in various physiological processes (6). In terms of the heart, lncRNAs have been shown to be closely related to the occurrence of heart disease (7). For example, lncRNA GAS5 is identified to be downregulated in the serum of AF patients and shows a strong association with the progression and recurrence of AF (8). Another lncRNA LICPAR is also reported to promote the viability and proliferation of AFs and is involved in the progress of AF *via* modulating TGF-β/Smad pathway (9). Taurine upregulated gene 1 (TUG1), an evolutionarily conserved lncRNA, has been recently reported to be linked to several heart diseases (10). In a recent study, TUG1 is reported to be induced in ischemia challenged cardiomyocytes, and downregulation of TUG1 may be a new target against the injury of acute myocardial infarction (AMI) (11). Silencing TUG1 is also suggested to inhibit cardiac hypertrophy, which is considered to be the major risk factor for the occurrence of AF (10, 12). Moreover, in a study about fibrotic deterioration evoked by chronic hypoxia, TUG1 is reported to promote cardiac fibroblast–myofibroblast transformation (FMT) activation and contribute to fibrosis in chronic hypoxia (13). However, the role of lncRNA TUG1 in AF has not been eliminated.

In recent years, lncRNAs have gradually become the focus of research worldwide, and the aberrant expression of lncRNAs can regulate the gene expression interacting with microRNAs (14). Both lncRNAs and microRNAs play a key role in the regulation of cell growth and apoptosis (15). In the study of cardiac hypertrophy, TUG1 is identified to inhibit the expression of miR-29b-3p, and TUG1 contributes to cardiac hypertrophy *via* sponging miR-29b-3p (10). In particular, miR-29b-3p has been considered to be an antifibrotic factor, and overexpression of miR-29b-3p may inhibit cardiac fibrosis and systemic sclerosis (16). Therefore, the current study detected the levels of TUG1 in AF patients. In addition, from the perspective of competing endogenous RNA (ceRNA) regulatory mechanism, the present study further explored the role of lncRNA TUG1 in the proliferation of CF and its underlying mechanism under the involvement of miR-29b-3p.

## Materials and Methods

### Study Subjects

A total of 104 patients with paroxysmal AF were recruited, who were admitted to Changle People's Hospital between August 2018 and January 2020. All AF patients were diagnosed according to the 2011 ACC/AHA/ESC guidelines (17). The exclusion criteria were as follows: cardiomyopathy, congenital heart disease, valvular disease, chronic inflammatory diseases, autoimmune connective tissue disease, diseases of blood system, hyperthyroidism, malignant tumor, severe liver and kidney function impairment, history of cardiac surgery, a history of severe trauma or surgery within 2 months, and glucocorticoid administration within 1 month. In addition, a group of 94 age- and gender-matched healthy individuals were collected as the control group. All participants underwent clinical evaluation, blood routine examination, and echocardiographic examination. Data on demographics including gender, age, and body mass index (BMI) were collected from the hospital database. The resting heart rate (RHR) of paroxysmal AF patients in sinus rhythm was recorded. RHR was measured three times for each individual in a sitting position with 5 min interval, and the mean of readings was used.

The design and protocol of this experiment were approved by the Ethics Committee of the Changle People's Hospital. Study subjects were recruited on a volunteer basis, and their written informed consents were received before participation.

### Sample Collection

Fasting venous blood samples (6 ml) were collected from each subject in the early morning. After resting for 10 min at room temperature, the blood samples were centrifuged for 15 min, and then the serum samples were collected and stored at −80°C for subsequent experiments.

### Echocardiographic Measurements

All echocardiographic data were obtained during sinus rhythm in both groups. Measurements of left atrial diameter (LAD), left ventricular diameter (LVD), left ventricular wall thickness (LVWT), and left ventricular ejection fraction (LVEF) were performed according to the guidelines of the American Society of Echocardiography (ASE). Left atrial volume index (LAVI) was defined as the ratio of the LA volume to the body surface area. Doppler echocardiography was performed to obtain transmitral inflow images for measurement of the peak E and A wave velocities, as well as the ratio of these velocities (E/A). The deceleration time (DCT; the time between peak E and the intersection of the upper deceleration slope and the zero baselines) was also measured. Diastolic dysfunction was classified into three levels (abnormal relaxation: E/A <0.75 or DCT >240 ms; pseudo-normal LV filling: E/A 0.75–1.50, DCT 151–240 ms, and LA volume ≥28 ml/m^2^; restrictive diastolic filling: E/A >1.5 or DCT ≤ 150 ms), as defined previously (18).

### Cell Culture and Treatment

The human cardiac fibroblasts (HCFs) were provided by ScienCell Research Laboratory (San Diego, CA). All cells were cultured in Dulbecco's modified Eagle's medium (DMEM; Thermo Fisher Scientific, Waltham, MA, USA) supplemented with 10% fetal bovine serum (FBS; Invitrogen Life Technologies, Carlsbad, CA, USA) and penicillin–streptomycin solution in an incubator with 5% CO_2_ and 95% air at 37°C. The HAFs were treated with 10^−7^ mol/L angiotensin II (AngII; Sigma-Aldrich, St. Louis, MO, USA) for 24 h to establish a cell model of AF as previously reported (19).

To regulate the gene expression level, cells were transfected with TUG1 siRNA (si-TUG1) or its negative control (si-NC) and with miR-29b-3p inhibitor (anti-miR-29b-3p) or its negative control (anti-NC) before AngII treatment, which were purchased from RiboBio (Guangzhou, China). In brief, si-TUG1, si-NC, anti-miR-29b-3p, or anti-NC (50 nmol/L) was transfected into logarithmic growth HAFs by using Lipofectamine 2000 and incubated in DMEM for 24 h before AngII treatment.

### RNA Exaction and qRT-PCR

Total RNA was extracted by using TRIzol reagent (Invitrogen; Thermo Fisher Scientific, Inc.), and the RNA purity was detected through OD_260_/OD_280_ measurement. For miR-29b-3p expression analysis, the RNA was first reversely transcribed into cDNA using miRNA cDNA Synthesis Kit (Cwbiotech, Beijing, China), and then miRNA qPCR Assay Kit (Cwbiotech, Beijing, China) was used for the quantitation of miR-29b-3p according to the manufacturer's instructions. For gene expression, RNA was reversely transcribed into cDNA using HiFiScript cDNA Synthesis Kit (Cwbiotech, Cwbiotech, Beijing, China). Then, the products were performed by qRT-PCR analysis by using UltraSYBR Mixture (Cwbiotech, Cwbiotech, Beijing, China) according to the manufacturer's instructions. The following thermocycling conditions were used for the PCR: initial denaturation at 94°C for 2 min, followed by 40 cycles of 94°C for 20 s, and 60°C for 34 s. To normalize RNA level, U6 was used for miR-29b-3p normalization and GAPDH was used for TUG1 and TGF-β1. The relative gene expression was calculated by using the 2^−Δ*ΔCT*^ method. The primer sequences were as follows: TUG1 forward (5′-TAGCAGTTCCCCAATCCTTG-3′), reverse (5′-CACAAATTCCCATCATTCCC-3′); GAPDH forward (5′-GAGTCAACGGATTTGGTCGT-3′), reverse (5′-TTGATTTTGGAGGGATCTCG-3′); miR-29b-3p forward (5′-TGCGGTAGCACCATTTGAAAT-3′), reverse (5′-CCAGTGCAGGGTCCGAGGT-3′); and U6 forward (5′-CCTGCTTCGGCAGCACA-3′), reverse (5′-AACGCTTCACGAATTTGCGT-3′).

### Cell Counting Kit-8 Assay

Cell proliferation was detected using Cell Counting Kit-8 (CCK-8) (Dojindo, Shanghai, China); 4 × 10^3^ logarithmic growth HAFs were seeded into 96-well plates and incubated in DMEM. After incubation at 0, 24, 48, and 72 h, each well was added into 10 μl of CCK-8, and cultured in a dark environment for another 2 h. Then, the optical density (OD) value at a wavelength of 450 nm was detected at different incubation times by a microplate reader (Bio-Rad).

### Luciferase Reporter Assay

Luciferase reporter assay was performed for target gene analysis. In brief, the wild type (WT) or mutant type (MUT) of TUG1 or TGF-β1 sequences were cloned into the luciferase reporter vector psiCHECK-2 (Promega Corporation) according to the manufacturer's instruction. Cells were plated into 24-well plates and co-transfected with 500 ng of each reporter construct (WT-TUG1, MUT-TUG1, WT-TGF-β1, or MUT-TGF-β1) and miR-29b-3p mimic (miR-29b-3p) or miR-29b-3p inhibitor (anti-miR-29b-3p). Post-incubation for 48 h, the luciferase activity of each group was detected using the Dual-Luciferase Reporter System (Promega Corporation, USA) according to the instructions of the manufacturer. Renilla fluorescence activity was identified as the internal reference.

### Statistical Analyses

All statistical analyses were conducted under GraphPad Prism software (GraphPad, La Jolla, CA, USA) or SPSS 18.0 statistical software package (SPSS, Chicago, USA). Each experiment was repeated at least three times, using triplicate parallel samples within each experiment, and the data were expressed as means ± standard deviations (SD). Pearson correlations were used to detect the correlation of different variables, and the variables were checked for normality *via* the Kolmogorov–Smirnov (K–S) normality test. Differences between the two groups were compared using Student's *t*-test, whereas one-way analysis of variance (ANOVA) analysis was applied for the comparison of multiple groups. Data followed by *p* < 0.05 were considered to be statistically significant.

## Results

### Demographics and Clinical Characteristics

As shown in Table 1, there was no significant difference in terms of age, gender, BMI, course of disease, and prevalence of smoking, hypertension, and diabetes between control and AF groups (*p* > 0.05). Based on the echocardiographic data, patients in the AF group had larger LAD, LVD, and LVWT, and higher LAVI and LVEF compared with the control group (*p* < 0.05). Higher level of RHR was detected in the AF group compared with the control group (*p* < 0.05), which might be related to an increase in cardiac sympathetic tone. The AF group patients had more severe diastolic dysfunction compared with the control group patients (*p* < 0.05). Medication of patients in the two groups was also shown in Table 1, but there was no significant difference between the two groups (*p* > 0.05).

**Table 1:** Clinical data of the study subjects.

**Parameter**	**Control group**	**AF patient group**	***P*-value**
	**(*n* = 94)**	**(*n* = 104)**	
Gender (male/female)	49/45	54/50	0.977
Age (years)	65.68 ± 6.71	65.06 ± 6.25	0.500
BMI (Kg/m^2^)	25.02 ± 2.53	25.67 ± 2.56	0.075
Smoking	27 (28.72%)	32 (30.77%)	0.753
Hypertension	48 (51.06%)	58 (55.77%)	0.507
Diabetes	16 (17.02%)	18 (17.31%)	0.957
Course of disease months)	–	16.16 ± 8.53	–
LAD (mm)	37.28 ± 3.51	40.77 ± 4.65	<0.001
LVD (mm)	46.96 ± 3.13	48.59 ± 5.63	0.014
LVWT (mm)	8.26 ± 1.79	9.03 ± 0.71	<0.001
LAVI (mL/m^2^)	18.13 ± 4.75	38.12 ± 10.63	<0.001
LVEF (%)	58.78 ± 3.14	57.28 ± 10.53	0.028
RHR	59.23 ± 5.47	69.99 ± 5.95	<0.001
Medicaments			
Beta blockers	30 (31.91%)	38 (36.54%)	0.494
ACE inhibitor	24 (25.53%)	30 (28.85%)	0.601
Sartans	15 (15.96%)	21 (20.19%)	0.440
Statins	5 (5.32%)	7 (6.73%)	0.678
Diastolic dysfunction			
Abnormal relaxation	47 (50.00%)	35 (33.65%)	0.020
Pseudo-normal LV filling	8 (8.51%)	20 (19.23%)	0.031
Restrictive diastolic	7 (7.45%)	16 (15.38%)	0.082

*AF, Atrial fibrillation; BMI, body mass index; LAD, left atrial diameter; LVD, left ventricular diameter; LVWT, left ventricular wall thickness; LAVI, left atrial volume index; LVEF, left ventricular ejection fraction; RHR, resting heart rate*.

### Aberrant Expression of TUG1 and miR-29b-3p in AF Patients

**Figure 1:**
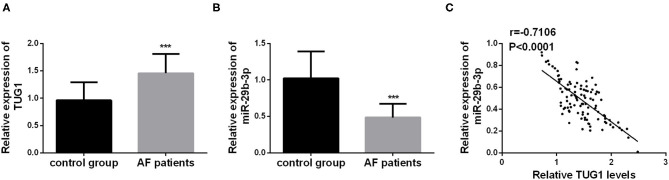
Aberrant expression of TUG1 and miR-29b-3p in AF patients. **(A)** TUG1 was highly expressed in the serum of AF patients compared with the healthy controls (***p < 0.001). **(B)** miR-29b-3p was downregulated in AF patients compared with the control group (***p < 0.001). **(C)** Serum TUG1 showed negative correlation with the level of miR-29b-3p in AF patients (*r* = −7.106, *p* < 0.001).

The serum levels of TUG1 and miR-29b-3p were detected by using qRT-PCR in each individual, and the values were compared between the AF and control groups using the Student's *t*-test. The statistical analysis results demonstrated that TUG1 was highly expressed in the serum of AF patients compared with the healthy controls, whereas downregulation of miR-29b-3p was detected in AF patients (Figures 1A,B, *p* < 0.001). Moreover, Pearson's correlation analysis exhibited an inverse relationship between TUG1 and miR-29b-3p expression in AF patients (*r* = −0.7106, *p* < 0.001; Figure 1C).

### Correlation of TUG1 and miR-29b-3p With Clinical Data in AF Patients

As shown in Table 2, positive associations were detected for serum TUG1 levels with echocardiographic data in the AF group, including LAD (*r* = 0.267, *p* = 0.006), LVD (*r* = 0.346, *p* < 0.001), LVWT (*r* = 0.494, *p* < 0.001), LAVI (*r* = 0.561, *p* < 0.001), LVEF (*r* = 0.369, *p* < 0.001), and RHR (*r* = 0.373, *p* < 0.001). Inversely, these echocardiographic data were negatively correlated with serum miR-29b-3p levels. It was noted that the positive correlations of serum TUG1 levels with echocardiographic data are generally small/modest (*r* < 0.5), except LAVI (*r* > 0.5). It might reflect the fact that LAVI is much more accurate in detecting atrial dimensions than LAD (20).

**Table 2:** Correlation of TUG1 and miR-29b-3p with various indicators respectively.

**Parameters**	**TUG1 (*r*)**	***P-value***	**MiR-29b-3p (*r*)**	***P-value***
LAD (mm)	0.267	0.006	−0.242	0.013
LVD (mm)	0.346	<0.001	−0.340	<0.001
LVWT (mm)	0.494	<0.001	−0.435	<0.001
LAVI (mL/m^2^)	0.561	<0.001	−0.523	<0.001
LVEF (%)	0.160	0.104	−0.107	0.281
RHR	0.373	<0.001	−0.329	0.001

*LAD, left atrial diameter; LVD, left ventricular diameter; LVWT, left ventricular wall thickness; LAVI, left atrial volume index; LVEF, left ventricular ejection fraction; RHR, resting heart rate*.

### Knockdown of TUG1 Inhibited AngII-Induced CF Proliferation

**Figure 2:**
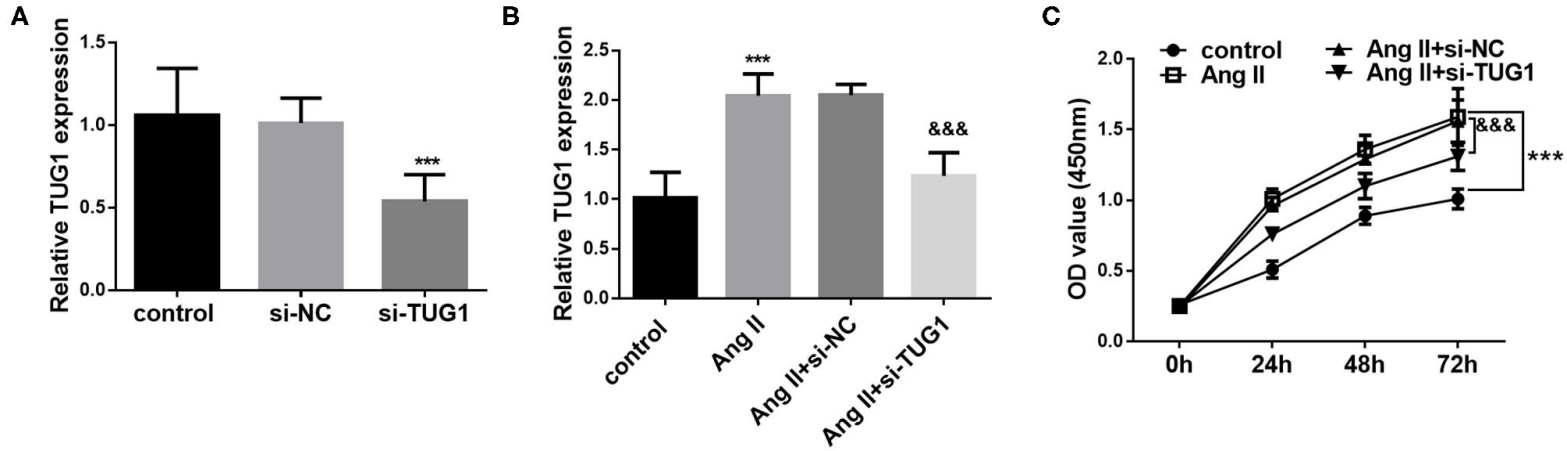
Knockdown of TUG1 inhibited AngII-induced CF proliferation. **(A)** si-TUG1 transfection significantly decreased the level of TUG1 in CFs. **(B)** AngII treatment significantly upregulated the level of TUG1, which was reversed by TUG1 knockdown. **(C)** AngII promoted CF proliferation, but knockdown of TUG1 reversed the promotion effect of AngII on CF proliferation (***p < 0.001, compared with the control group; ^&&&^*p* < 0.001, compared with the AngII group).

Considering the aberrant expression of TUG1 in AF patients, an AF cell model was established in human CFs by using AngII. As shown in Figure 2B, AngII treatment significantly upregulated the level of TUG1, which was consistent with the results found in clinical samples (*p* < 0.001). To further investigate the role of TUG1 in CF proliferation, its expression level was regulated *via* cell transfection. The qRT-PCR results indicated that si-TUG1 transfection significantly decreased the level of TUG1 in CFs (*p* < 0.001; Figure 2A) and reversed the upregulation of TUG1 induced by AngII (*p* < 0.001; Figure 2B). Results from Figure 2C indicated that AngII promoted CF proliferation, but knockdown of TUG1 reversed the promotion effect of AngII on CF proliferation (*p* < 0.001).

### TUG1 Functions as a ceRNA for miR-29b-3p

**Figure 3:**
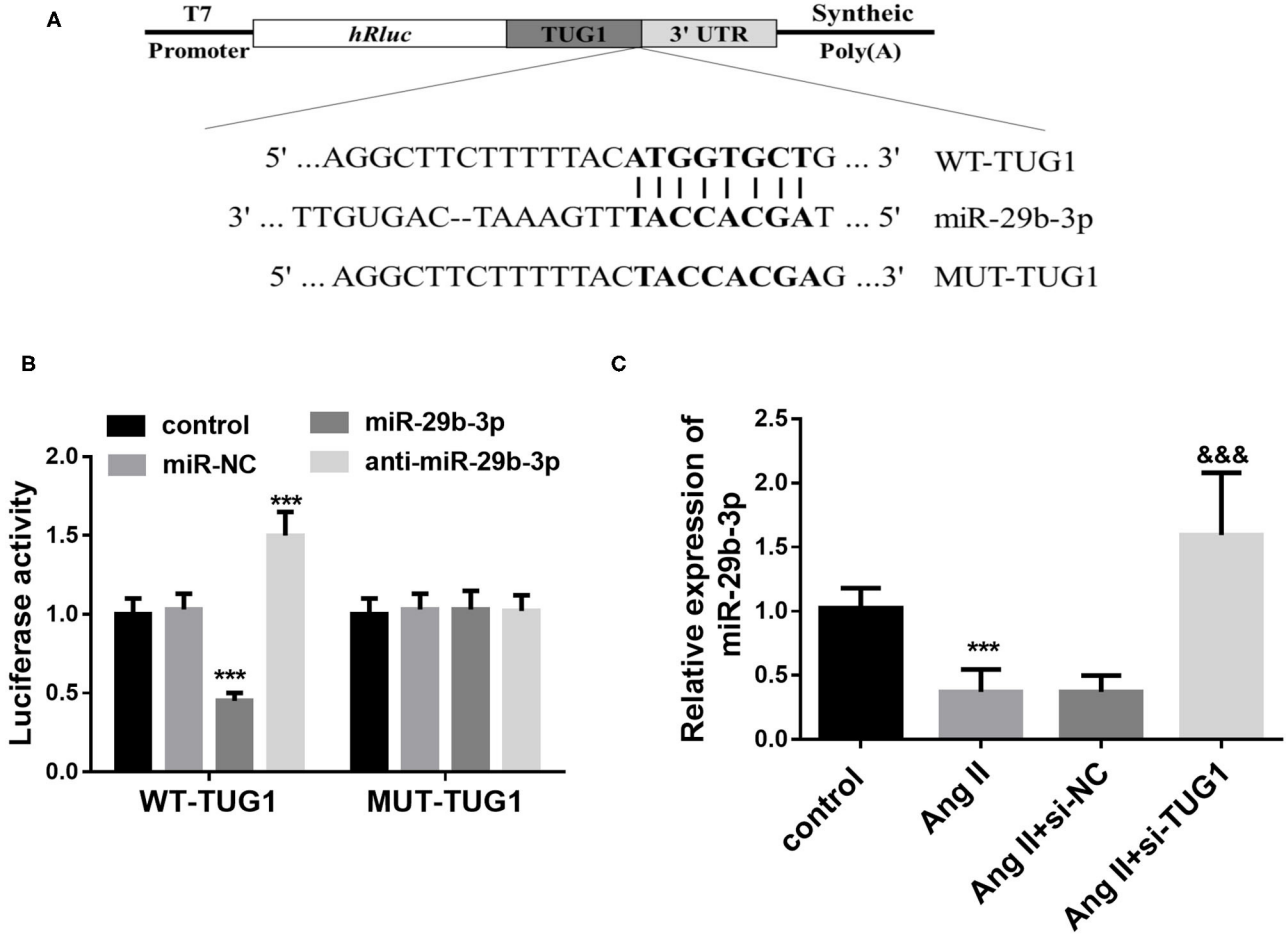
TUG1 functions as a ceRNA for miR-29b-3p. **(A)** TUG1 contains complementary sequences that bind miR-29b-3p. **(B)** Overexpression of miR-29b-3p inhibited the luciferase activity of CFs transfected with TUG1-WT, whereas miR-29b-3p enhanced the luciferase activity. miR-29b-3p showed no significant influence on the luciferase activity of CFs transfected with TUG1-WT vector. **(C)** In the AF cell model, a low expression of miR-29b-3p was observed in AngII-treated cells, and TUG1 knockdown upregulated the expression of miR-29b-3p (***p < 0.001, compared with the control group; ^&&&^*p* < 0.001, compared with the AngII group).

StarBase V3.0 analysis results indicated that TUG1 contains complementary sequences that bind miR-29b-3p (Figure 3A). To further verify whether TUG1 directly bound miR-29b-3p, a dual-luciferase reporter assay was performed by co-transfecting TUG1-WT or -MUT containing target sequences and miR-29b-3p in CF cells. It was observed that overexpression of miR-29b-3p inhibited the luciferase activity of CFs transfected with TUG1-WT, whereas miR-29b-3p enhanced the luciferase activity (*p* < 0.001; Figure 3B). However, miR-29b-3p showed no significant influence on the luciferase activity of CFs transfected with TUG1-WT vector (*p* > 0.05; Figure 3B). In addition, in the AF cell model, a low expression of miR-29b-3p was observed in AngII-treated cells, and TUG1 knockdown upregulated the expression of miR-29b-3p (*p* < 0.001; Figure 3C).

### Downregulation of miR-29b-3p Reversed the Role of TUG1 in CF Proliferation

**Figure 4:**
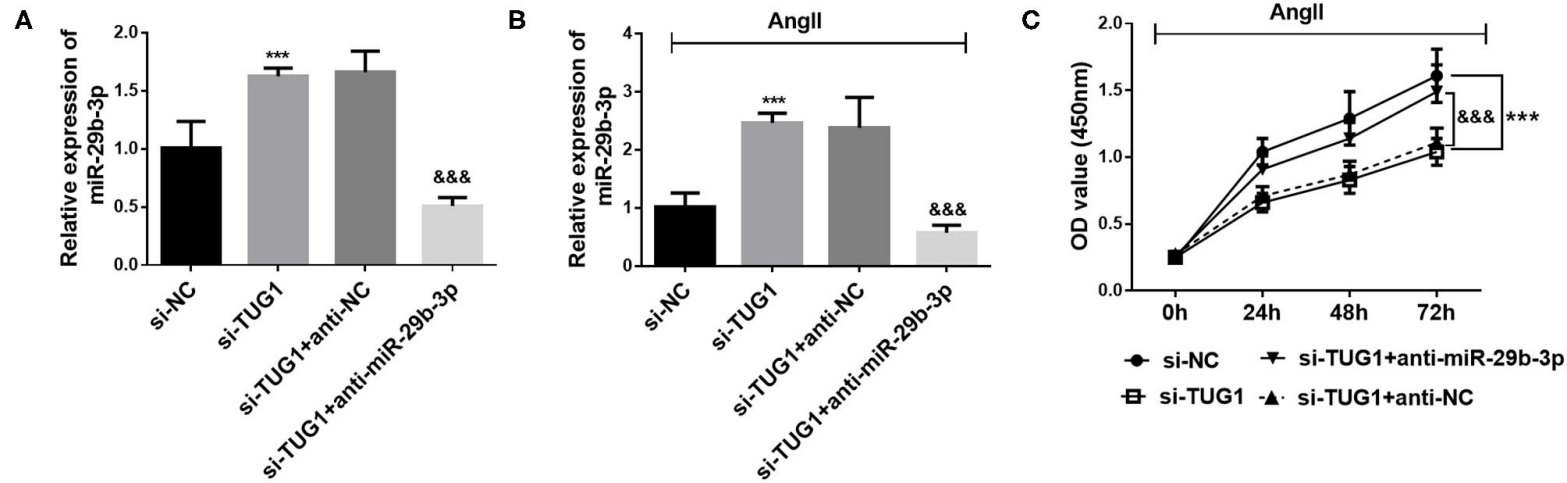
Downregulation of miR-29b-3p reversed the role of TUG1 in CF proliferation. **(A)** miR-29b-3p inhibitor transfection significantly reduced the level of miR-29b-3p in CFs (***p < 0.001). **(B)** miR-29b-3p downregulation significantly reversed the upregulation of miR-29b-3p induced by TUG1 knockdown (***p < 0.001, compared with the si-NC group; ^&&&^*p* < 0.001, compared with the si-TUG1 group). **(C)** Downregulation of miR-29b-3p reversed the inhibiting effect of TUG1 knockdown against CF proliferation (***p < 0.001, compared with the si-NC group; ^&&&^*p* < 0.001, compared with the si-TUG1 group).

To further investigate the interaction of miR-29b-3p and TUG1, the level of miR-29b-3p was downregulated by miR-29b-3p inhibitor transfection. It was observed that after miR-29b-3p inhibitor transfection, the level of miR-29b-3p was significantly decreased (*p* < 0.001; Figure 4A). Moreover, miR-29b-3p inhibitor transfection significantly reversed the upregulation of miR-29b-3p induced by TUG1 knockdown (*p* < 0.001; Figure 4B). Furthermore, downregulation of miR-29b-3p reversed the inhibiting effect of TUG1 knockdown against CF proliferation (*p* < 0.001; Figure 4C).

### TGF-β1 Is a Direct Target Gene of miR-29b-3p

**Figure 5:**
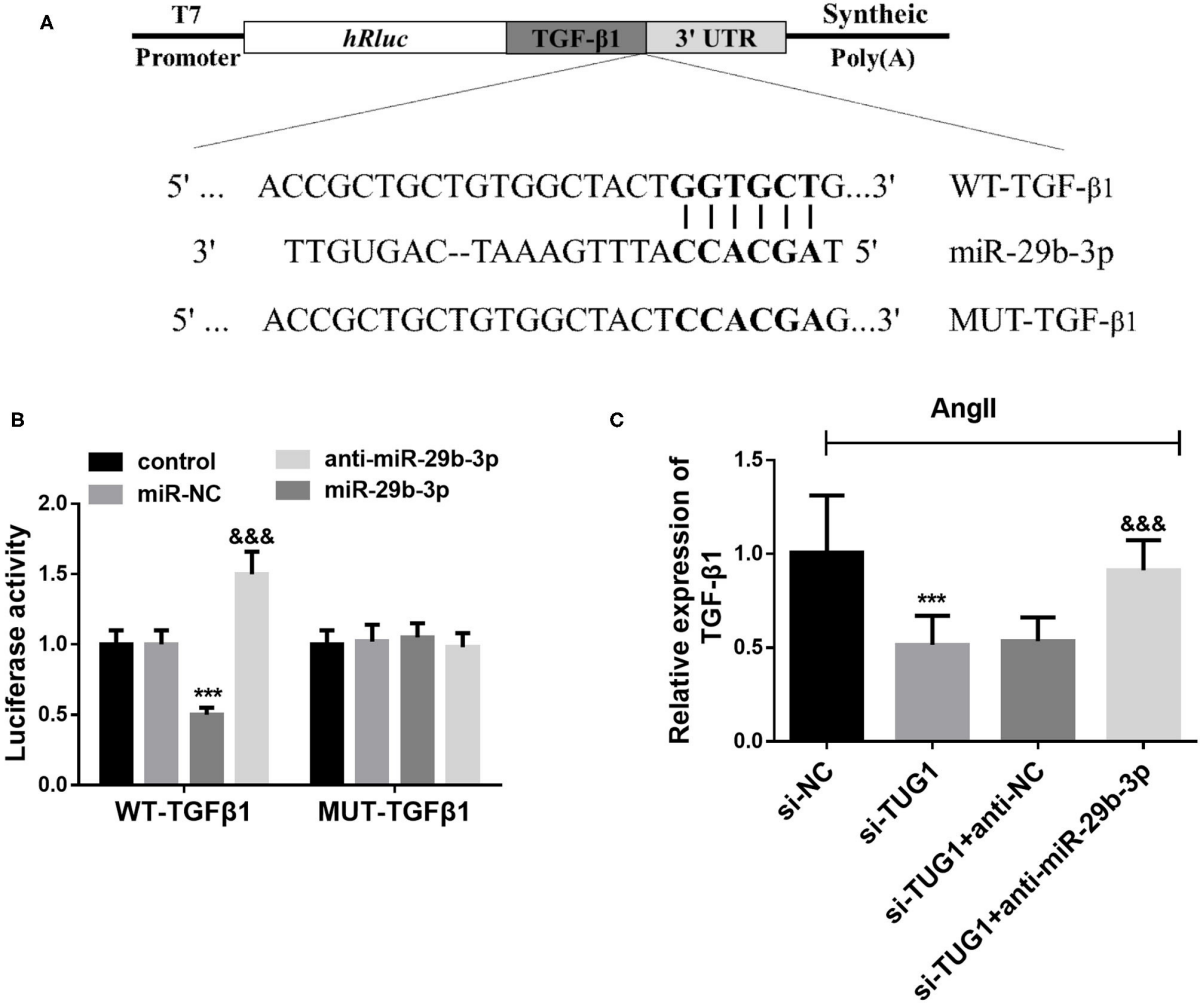
TGF-β1 is a direct target gene of miR-29b-3p. **(A)** Complementary sequences between miR-29b-3p and the 3′UTR of TGF-β1 according to the Target Scan analysis. **(B)** miR-29b-3p overexpression reduced the luciferase activity in TGF-β1-WT transfected cells, whereas miR-29b-3p downregulation strengthened the luciferase activity. For TGF-β1-MUT transfected cells. MiR-29b-3p expression did not influence the luciferase activity (***p < 0.001). **(C)** Downregulation of miR-29b-3p reversed the inhibiting effect of TUG1 knockdown on TGF-β1 expression in AF cell models (***p < 0.001, compared with the si-NC group; ^&&&^*p* < 0.001, compared with the si-TUG1 group).

Complementary sequences between miR-29b-3p and the 3′UTR of TGF-β1 were observed according to the Target Scan analysis results (Figure 5A). Then, the dual-luciferase reporter assay results demonstrated that miR-29b-3p overexpression reduced the luciferase activity in TGF-β1-WT transfected cells, whereas miR-29b-3p downregulation strengthened the luciferase activity (*p* < 0.001; Figure 5B). However, no significant changes were found for the luciferase activity of TGF-β1-MUT transfected cells no matter upregulating or downregulating miR-29b-3p (*p* > 0.05; Figure 5B). Besides, the qRT-PCR results demonstrated that downregulation of miR-29b-3p reversed the inhibiting effect of TUG1 knockdown on TGF-β1 expression in AF cell models (*p* < 0.001; Figure 5C).

## Discussion

Atrial fibrillation is a common arrhythmia in clinic, and its etiology and mechanism have been the focus of cardiovascular research. In the past few decades, most of the researches on the pathogenesis of diseases have focused on protein-coding genes (21). However, in recent years, with the application of new technologies and methods, more and more evidence shows that the pathological mechanisms of most diseases may be regulated by short or lncRNAs. According to the current researches, the aberrant expression of lncRNAs shows a close relationship with the occurrence of many tumors (22). In recent years, the roles of lncRNAs in cardiovascular diseases also attract attention (23). Long non-coding RNA taurine upregulated gene 1 (LncRNA TUG1) is located on chromosome 22q12.2, with a length of 7.1 kb, and it was initially detected in taurine-treated mouse retinal cells (24). Recent studies have found that TUG1 is involved in the development of several cardiovascular diseases, including aortic valve calcification, myocardial ischemia–reperfusion injury, myocardial infarction, and other cardiovascular diseases (25). The present study results indicated that TUG1 was overexpressed in the serum of AF patients and positively correlated with echocardiographic data, especially LAVI, which might reflect the fact that LAVI is much more accurate in detecting atrial dimensions than LAD (20). A positive association was also observed for serum TUG1 with RHR, which might be related to an increase in cardiac sympathetic tone of the patients. In addition, the aberrant expression of TUG1 has been reported to promote cardiac FMT activation and contribute fibrosis under chronic hypoxia conditions (13), reflecting its crucial role in myocardial fibrosis. Considering that, we hypothesized that higher serum levels of TUG1 might be associated with a high probability of progression to persistent and permanent AF.

Myocardial fibrosis plays a key role in the occurrence and development of AF. The link between CF proliferation and AF is now universally recognized, and overproliferation of CFs contributes to the increase of dysfunctional extracellular matrix (ECM) (26). In the present study, human CFs were treated with AngII to establish a cell model of AF as previously reported (19). Consistent with the results observed in clinical samples, high expression of TUG1 was also detected in the AF cell models. Furthermore, the loss and gain function experiments demonstrated that TUG1 knockdown inhibited CF proliferation. Consistently, TUG1 has been reported to promote cardiac FMT activation and contribute to fibrosis under chronic hypoxia conditions (13). In addition, TUG1 knockdown is also suggested to attenuate AngII-induced cardiac hypertrophy, which is considered to be the major risk factor for the occurrence of AF (10). All evidence supported our results about the important role of TUG1 in AF.

miRNA is an important regulatory factor in development, immune regulation, and other processes, and plays a key regulatory role in cardiovascular diseases (27). Accumulating evidence has demonstrated that miRNA expression levels are associated with the occurrence and severity of AF (28). The interaction between lncRNAs and microRNAs has been widely reported in many literatures (29). In a study of cardiac hypertrophy, TUG1 is reported to be a positive modulator of cardiac hypertrophy *via* sponging miR-29b-3p (10). Consistent with the previous study, the present study confirmed that lncRNA TUG1 functions as a ceRNA for miR-29b-3p in AF. Moreover, Pearson's correlation analysis revealed an inverse relationship between TUG1 and miR-29b-3p expression in clinical serum samples. Consistently, low expression of miR-29b-3p was also detected in CFs treated with AngII. In a study of microRNA expression signatures of AF, reduced miR-29b-3p in AF patients was identified by using bioinformatics (30). Another study also suggests a low expression of miR-29b-3p in the plasma of paroxysmal AF patients (31). Consistent with these reports, the present study confirmed that miR-29b-3p was downregulated in the serum of AF patients and negatively associated with the level of serum TUG1 levels. Furthermore, the *in vitro* experiment results indicated that downregulation of miR-29b-3p reversed the inhibiting effect of TUG1 knockdown against CF proliferation. According to these data, we concluded that TUG1 regulates the proliferation of CFs *via* targeting miR-29b-3p. Consistently, in a study of aortic stenosis (AS), downregulation of miR-29b-3p in fibroblasts is considered to be the mechanism underlying the fibrotic effect of TGF-β in the stressed left ventricle myocardium (32). These findings supported our speculation about the crucial role of TUG1/miR-29b-3p in myocardial fibrosis, even in the progression of paroxysmal AF to persistent and permanent AF. In addition, considering the aberrant expression of TUG1/miR-29b-3p in the serum of AF patients, it will be interesting to investigate these expressions in the culture medium in which CFs were treated by angiotensin. However, in the current study, only the levels in cells were detected, and it is of great significance for further exploration.

TGF-β1 is a multifunctional cytokine involved in cell proliferation, apoptosis, and migration. It is known that overexpression of TGF-β1 can lead to cardiac fibrosis (33). In transgenic mouse models, overexpression of TGF-β1 was observed in mice with lone AF (34). The inhibitory effect of the anti-fibrosis drug pirfenidone on TGF-β1 can significantly reduce the degree of atrial fibrosis (35). These studies suggest that TGF-β1 plays an important role in the atrial fibrosis of AF patients. Consistently, a high level of TGF-β1 is also observed in the atrial tissues of AF cases and associated with the occurrence of arrhythmia events (36). These studies further demonstrate that AF is positively correlated with TGF-β1 levels. The results of the present study further indicated that TGF-β1 is a direct target of miR-29b-3p in AF. Because of the crucial role of TGF-β1 in AF, we hypothesized that lncRNA TUG1 is involved in the development of AF by regulating the miR-29b-3p/TGF-β1 axis. However, further studies are needed to confirm the regulation between TGF-β1 and TUG1/miR-29b-3p in AF, and its potential regulatory mechanism should be examined in the future.

In conclusion, the present results demonstrated that lncRNA TUG1 is a key regulator in the occurrence of AF and may be involved in the atrial fibrosis in AF patients. Functionally, slicing TUG1 inhibits CF proliferation by regulating the miR-29b-3p/TGF-β1 axis. The data affirm the potential role of TUG1/miR-29b-3p in AF therapy and provide the theoretical basis for the mechanistic understanding of AF.

## Data Availability Statement

The raw data supporting the conclusions of this article will be made available by the authors, without undue reservation.

## Ethics Statement

The studies involving human participants were reviewed and approved by the design and protocol of this experiment were approved by the Ethics Committee of the Changle People's Hospital. The patients/participants provided their written informed consent to participate in this study.

## Author Contributions

All authors participated in this study. YG participated in the design of this study. ZS performed the statistical analysis. JL carried out the study and collected important background information. MC drafted the manuscript.

## Conflict of Interest

The authors declare that the research was conducted in the absence of any commercial or financial relationships that could be construed as a potential conflict of interest.

## Publisher's Note

All claims expressed in this article are solely those of the authors and do not necessarily represent those of their affiliated organizations, or those of the publisher, the editors and the reviewers. Any product that may be evaluated in this article, or claim that may be made by its manufacturer, is not guaranteed or endorsed by the publisher.
